# Assessing the nutritional quality of *Pleurotus ostreatus* (oyster mushroom)

**DOI:** 10.3389/fnut.2023.1279208

**Published:** 2024-01-16

**Authors:** Magdalene Eno Effiong, Chidinma Precious Umeokwochi, Israel Sunmola Afolabi, Shalom Nwodo Chinedu

**Affiliations:** ^1^Department of Biochemistry, College of Science and Technology, Covenant University, Ota, Nigeria; ^2^Covenant Applied Informatics and Communication Africa Centre of Excellence (CApIC-ACE), Covenant University, Ota, Ogun, Nigeria; ^3^Covenant University Public Health and Wellbeing Research Cluster (CUPHWERC), Covenant University, Ota, Nigeria

**Keywords:** *Pleurotus* species, minerals, vitamins, proximate, amino acids, functional foods, human health

## Abstract

There is a huge gap between food production and the exploding population demands in various parts of the world, especially developing countries. This increases the chances of malnutrition, leading to increased disease incidence and the need for functional foods to reduce mortality. *Pleurotus ostreatus* are edible mushrooms that are cheaply sourced and rich in nutrient with the potential to be harnessed toward addressing the present and future food crisis while serving as functional foods for disease prevention and treatment. This study evaluated the nutritional, proximate, vitamins and amino acids contents of *Pleurotus ostreatus*. The proximate composition of *Pleurotus ostreatus* in this study revealed that it contains 43.42% carbohydrate, 23.63% crude fiber, 17.06% crude protein, 8.22% ash, 1.21% lipid and a moisture content of 91.01 and 6.46% for fresh and dry samples of *Pleurotus ostreatus, respectively*. The monosaccharide and disaccharide profile of *Pleurotus ostreatus* revealed the presence of glucose (55.08 g/100 g), xylose (7.19 g/100 g), fructose (19.70 g/100 g), galactose (17.47 g/100 g), trehalose (7.37 g/100 g), chitobiose (11.79 g/100 g), maltose (29.21 g/100 g), sucrose (51.60 g/100 g) and lower amounts of cellobiose (0.01 g/100 g), erythrose (0.48 g/100 g) and other unidentified sugars. Potassium, Iron and Magnesium were the highest minerals present with 12.25 mg, 9.66 mg and 7.00 mg amounts, respectively. The vitamin profile revealed the presence of vitamin A (2.93 IU/100 g), C (16.46 mg/100 g), E (21.50 mg/100 g) and B vitamins with vitamin B2 having the highest concentration of 92.97 mg/kg. The amino acid scores showed that *Pleurotus ostreatus* had more non-essential amino acids (564.17 mg/100 g) than essential amino acids (67.83 mg/100 g) with a ratio of 0.11. Lysine (23.18 mg/100 g) was the highest essential amino acid while aspartic acid (492.12 mg/kg) was the highest non-essential amino acid present in *Pleurotus ostreatus*. It had a higher concentration of acidic amino acids, 492.12 mg/100 g (77.87%), followed by neutral amino acids, 106.66 mg/100 g (16.88%) and least were the basic amino acids, 23.18 mg/100 g (3.67%). Based on the nutritional assessment of the *Pleurotus ostreatus* analyzed in this study, it can be concluded that it can serve as an important functional food source that can be exploited to meet the increasing food demands and reduce micronutrient deficiencies in many parts of the world, especially developing countries.

## Introduction

Nutrition plays an indispensable role in the sustenance of life (1). Plants and animals have been relied upon as vital sources of food and raw materials that play important roles in maintaining food security and preventing malnutrition (2). These nutritive compounds include lipids/fats, proteins, fiber, carbohydrates, minerals and vitamins which are highly essential for the proper functioning of the body (3).

However, the current world population explosion has been estimated to increase to 10 billion by the year 2050 (4). This population increase, especially in Africa, has led to the possibility of food shortages, micronutrient deficiencies, poor nutrition and high consumption of unhealthy junk foods with numerous preservatives (5). These contribute to an increase in susceptibility, incidence and mortality from communicable and non-communicable diseases (6). It has also given rise to the search for alternative food sources which are readily available, cheaply sourced and nutritionally loaded.

Functional foods are substances that are rich in nutrient and confer additional health benefits when consumed (7). They often contain essential nutrients and bioactive compounds that help the body balance its nutrient deficiencies, boasts its immunity and reduce free radical generation thereby preventing oxidative stress and diverse nutrition-related diseases (8). Numerous naturally occurring foods have been found to have functional properties when consumed or utilized as food addictive, however, numerous constraints such as seasonal variations, cost of production or cultivation and availability hinder their utilization and constant supply.

Mushrooms are highly unexploited resources of the world belonging to the kingdom of fungi (9). They are primarily decomposers and active agents of bioremediation in the ecosystem. They do not contain chlorophyll and obtain their nutrients from the metabolism of non-living matter (10). They serve as a viable source of food loaded with nutraceuticals and have excellent organoleptic properties. The poisonous nature of most mushrooms has undermined its use as a source of food. However, various *Pleurotus* species of mushrooms are edible and highly nutritious (11).

*Pleurotus* species such as *Pleurotus ostreatus* thrive well on rotten materials and are cultivated in a simple and cheap way (12). They possess the ability to serve as functional foods and contribute richly to the nutritious value of food when used as food addictive due to their high content of proteins, carbohydrate, minerals, vitamins, antioxidants, phytochemicals and low fat content (13). Their rich carbohydrate content consists of a wide array of oligosaccharides, ergothioneine, mono- and disaccharides which perform prebiotic functions and are highly beneficial in maintaining a healthy gut microbiome (14, 15). They are sources of quality protein that supply essential amino acids to the body making them a suitable alternative to red meat and other animal proteins, especially for vegetarians (16). As a result, there is a need to ascertain the nutritional value of *Pleurotus ostreatus* with the aim of validating its nutrient composition to qualify as a functional food. The aim of this study is to evaluate the proximate, vitamins, minerals and amino acids composition of *Pleurotus ostreatus* to determine its suitability as a functional food.

## Materials and methods

### Materials

#### Sample collection

**Figure 1:**
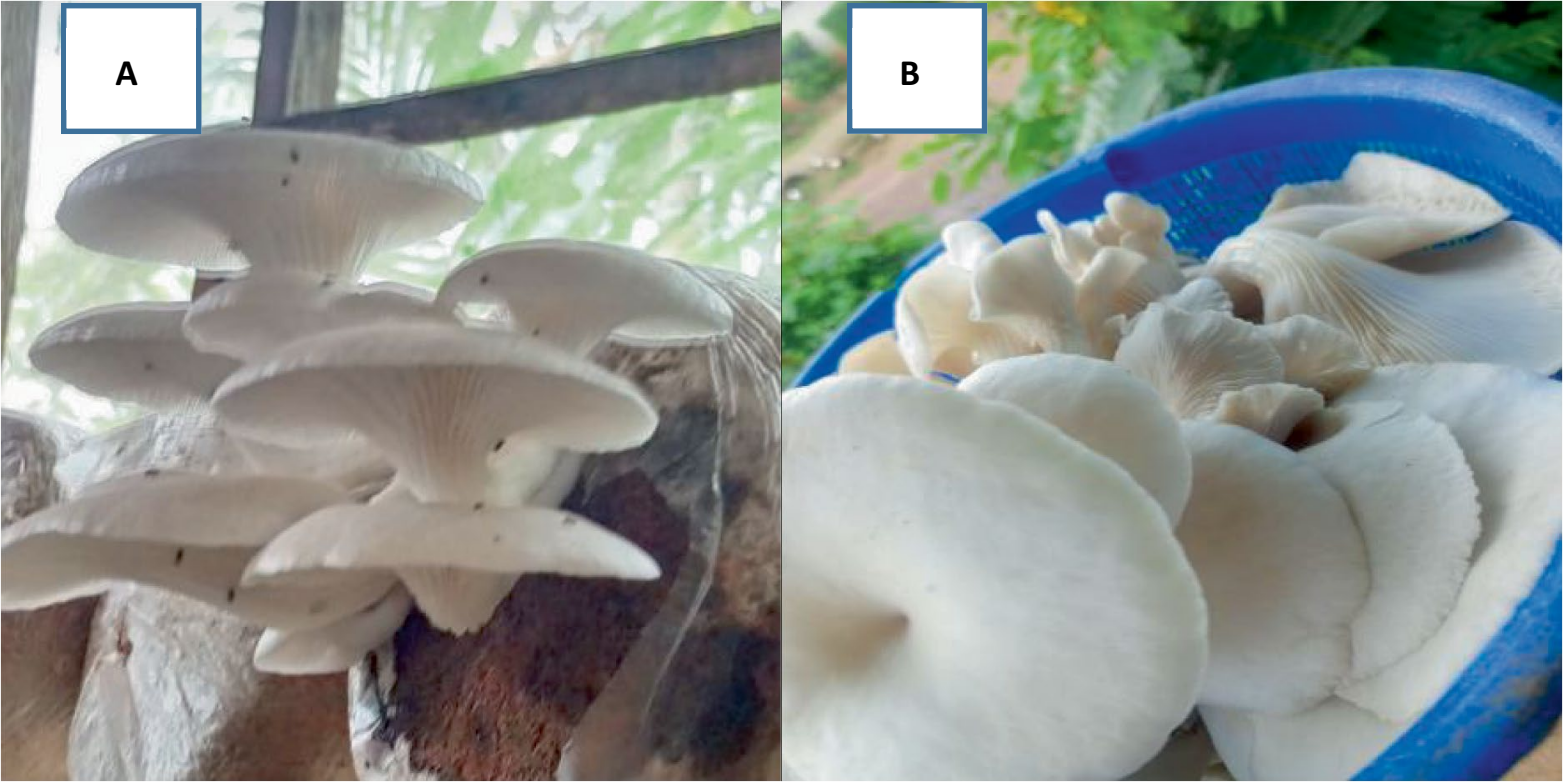
**(A,B)** *Pleurotus ostreatus* mushroom. **(A)** *Pleurotus ostreatus* growing on its substrate. **(B)** *Pleurotus ostreatus* freshly harvested.

Twenty-five kilograms (25 kg) of fresh *Pleurotus ostreatus*, oyster mushrooms were purchased from local mushroom farms in Agbara, Ogun state, South West, Nigeria, within the co-ordinates of 6.5114^o^N, 3.1115°E. It was grown on a combination of rice straw and saw dust as substrates. The specie purchased was authenticated by the botany department, University of Ibadan, Ibadan, Oyo state (Figure 1).

### Methods

#### Sample preparation

*Pleurotus ostreatus* was washed to remove impurities and wiped neatly with sterile cloth to remove water traces on its surface. The washed *Pleurotus ostreatus* was dried using hot-air in an oven at 55–65°C till completely dried. The dried *Pleurotus ostreatus* was then ground to powder using a blender and weighed. The resulting powder obtained was 2.5 kg which was cooled to room temperature and stored in air tight containers for further use (17).

#### Nutritional composition

The nutritional composition assessed in this study were proximate, carbohydrate profile, mineral element, vitamins and amino acids analysis.

#### Proximate analysis

The proximate composition analysis was carried out to assess the percentage crude fat, crude fibre, crude protein, ash, dry matter, moisture, calorific value and carbohydrate present in *Pleurotus ostreatus* according to the method described by AOAC (18), and Chinedu and Nwinyi (19).

#### Vitamin analysis

*Pleurotus ostreatus* was analyzed for vitamins A, B’s, C and E using the method described by Majesty et al. (2) and Sarwar et al. (20).

#### Determination of mineral elements

The prepared samples of *Pleurotus ostreatus* were analyzed for Iron (Fe), Zinc (Zn), Calcium (Ca), Magnesium (Mg), Sodium (Na), Potassium (K), Lead (Pb), Cadmium (Cd), Chromium (Cr), Nickel (Ni) using spectrophotometric methods according to Afolabi et al. (21) and AOAC (18).

#### Determination of amino acids

The amino acid content of the oyster mushrooms was determined using the method described by Varinsky et al. (22).

### Statistical analysis

Experiments were carried out in duplicates. Statistical analysis were carried out using means and standard deviation and graphical presentation was done using Microsoft excel.

## Results

### Proximate analysis of *Pleurotus ostreatus*

The proximate analysis of *Pleurotus ostreatus* revealed the percentage content of carbohydrate, moisture, ash, lipid, crude fiber and crude protein. The results showed that the carbohydrate content was highest (43.42 ± 0.01%), followed by crude fiber (23.63 ± 0.01%), crude protein (17.06 ± 0.17), ash (8.22 ± 0.04%) and moisture (6.46 ± 0.04%) as the least (Table 1).

**Table 1:** Proximate content of *Pleurotus ostreatus.*

Proximate profile	Amount
Moisture (fresh mushroom) (%)	91.01 ± 0.08
Moisture (Dry mushroom) (%)	6.46 ± 0.04
Ash (%)	8.22 ± 0.04
Carbohydrate (%)	43.42 ± 0.01
Calorific value (Kj/100 g)	1199.08 ± 1.77
Lipid (%)	1.21 ± 0.02
Crude fiber (%)	23.63 ± 0.01
Crude protein (%)	17.06 ± 0.17

#### Monosaccharide and disaccharide composition of *Pleurotus ostreatus*

The monosaccharide and disaccharide composition of *Pleurotus ostreatus* is shown in Table 2. The monosaccharides present in *Pleurotus ostreatus* were erythrose, glucose, xylose, fructose and galactose. Glucose was the highest monosaccharide present with a concentration of 55.09 g/100, this is followed by fructose (19.70 g/100 g), galactose (17.47 g/100 g), xylose (7.19 g/100 g) and erythrose (0.48 g/100 g) as the least. Nine disaccharides were present in *Pleurotus ostreatus*. Sucrose was the highest disaccharide present with a concentration of 51.60 g/100 g, followed by maltose (29.21 g.100 g), chitobiose (11.9 g/100 g), trehalose (7.37 g/100 g) and cellobiose (0.0068 g/100 g). Four unidentified disaccharides were present, however, their concentrations were within the range of 0.0006–0.0068 g/100 g.

**Table 2:** Monosaccharide and disaccharide composition of *Pleurotus ostreatus* (Chromatograms are shown in Figures 2A,B).

Monosaccharides (Figure 2A)	Concentration (g/100 g)
Erythrose	0.4785
Glucose	55.0888
Xylose	7.1869
Fructose	19.6984
Galactose	17.4660
	
Disaccharides (Figure 2B)	Concentration (g/100 g)
Unidentified	0.0006
Unidentified	0.0045
Unidentified	0.0037
Cellobiose	0.0068
Trehalose	7.3733
Chitobiose	11.7858
Maltose	29.2144
Sucrose	51.6018
Unidentified	0.0066

Monosaccharides: LOD – 0.015-0.115 μg/mL; LOQ – 0.120-0.551 μg/mL.

Disaccharides: LOD – 0.018-0.125 μg/mL; LOQ – 0.126-0.559 μg/mL.

LOD, Limit of Detection; LOQ, Limit of Quantification.

**Figure 2:**
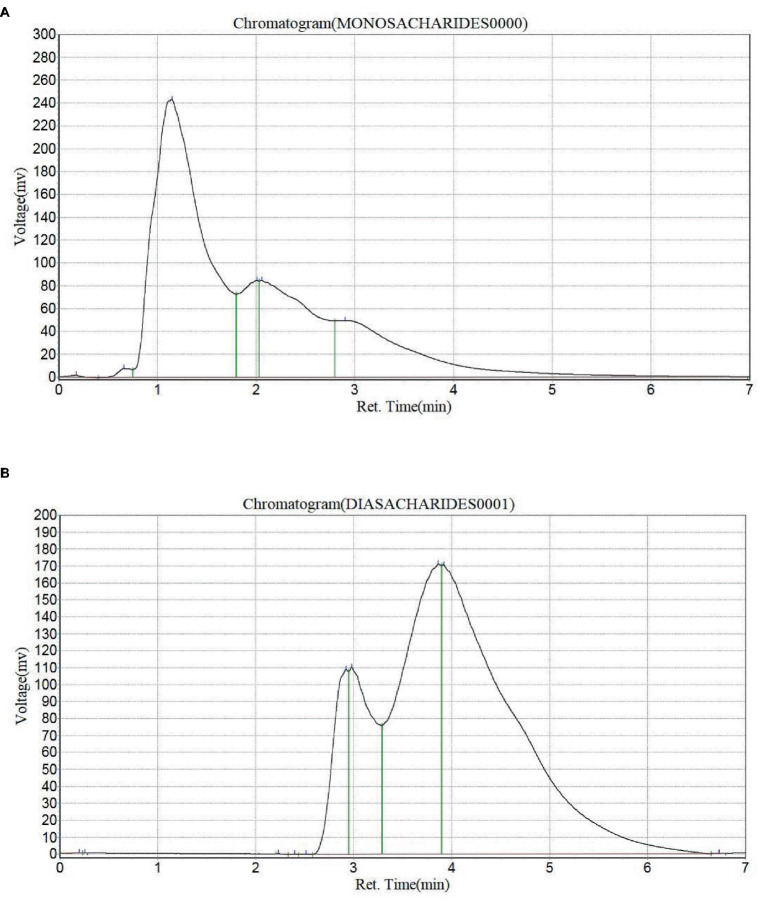
**(A)** Chromatogram for the monosaccharide profile of *Pleurotus ostreatus* and **(B)** chromatogram for the disaccharide profile of *Pleurotus ostreatus*.

#### Amino acid profile of *Pleurotus ostreatus*

**Figure 3:**
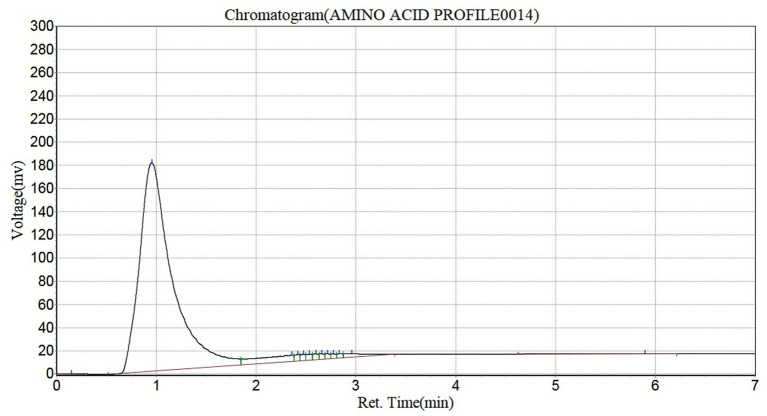
Chromatogram for the amino acid profile of *Pleurotus ostreatus.*

The amino acid profile of *Pleurotus ostreatus* is shown in Table 3. The results showed that it contained 13 amino acids, consisting of 5 essential amino acids and 8 non-essential amino acids. The amino acids present were leucine, threonine, methionine, phenylalanine, lysine, alanine, aspartic acid, proline, serine, asparagine, hydroxyproline, cysteine and glutamine. The highest amino acid present in *Pleurotus ostreatus* was aspartic acid (492.12 mg/100 g) while the least amino acid was cysteine (9.32 mg/100 g). The total essential amino acid concentration was 67.83 mg/100 g, with lysine (23.18 mg/100 g) having the highest concentration while the total non-essential amino acid was 564.17 mg/100 g, with aspartic acid (492.12 mg/100 g) having the highest concentration (Figure 3).

**Table 3:** Amino acid profile of *Pleurotus ostreatus.*

Amino acids	Conc (mg/100 g)	FAO/WHO/UNU (1981) (mg/g)
EAA		
Leucine	13.5670	70
Threonine	10.4144	40
Methionine	10.3644	23
Phenylalanine	10.3028	50
Lysine	23.1824	55
Total EAA	67.831	238
Non-EAA		
Alanine	10.0342	–
Aspartic acid	492.1208	–
Proline	10.4191	–
Serine	10.4585	–
Asparagine	10.4385	–
Hydroxyproline	9.3368	–
Cysteine	9.3234	–
Glutamine	12.0376	–
Total non-EAAs	564.1689	
Total Amino acids	632.00	

EAA* – Essential amino acid; non-EAA* – non-essential amino acid.

LOD – 0.105–1.812 ng/mL; LOQ – 0.210–2.501 ng/mL.

LOD, Limit of Detection; LOQ, Limit of Quantification.

The amino acid characteristics of *Pleurotus ostreatus* is shown is Table 4. The total amino acid concentration was 632.00 mg/100 g. The percentage essential amino acid content was 10.73% while the non-essential amino acid was 89.27%. The total acidic amino acid content (which consists of aspartic acid and glutamic acid) was 492.121 mg/100 g (77.87%), with aspartic acid as the only acidic amino acid present. The ratio of the essential amino acids (67.83 mg/100 g) to the total amino acids (632.00 mg/100 g) present in *Pleurotus ostreatus* was 0.11. Lysine was the only basic amino acid present out of the three basic amino acids (Arginine, lysine and Histidine) giving a total basic amino acid content of 23.18 mg/100 g (3.67%). The ratio of basic amino acids (23.18 mg/100 g) to acidic amino acids (492.121 mg/100 g) was 0.05. Only 11 neutral amino acids were present out of the 15 neutral amino acids in proteins (glycine, alanine, leucine, isoleucine, valine, phenyalaine, proline, methionine, serine, threonine, tyrosine, cysteine, glutamine, asparagine and tryptophan). The total concentration of the 11 neutral amino acids present in *Pleurotus ostreatus* was 106.66 mg/100 g (16.88%) consisting of leucine, threonine, methionine, phenylalanine, alanine, proline, serine, asparagine, hydroxyproline, cysteine and glutamine. The sulphur containing amino acids present in *Pleurotus ostreatus* was cysteine and methionine, making up a total concentration of 19.69 mg/100 g (3.12%). The percentage cysteine present in the sulphur containing amino acids was 47.36%. Out of the three branched amino acids (leucine, isoleucine and valine) present in proteins, only leucine was present with a concentration of 13.57 mg/100 g (2.15%). Phenylalanine was the only aromatic amino acid present in *Pleurotus ostreatus* compared to the four aromatic amino acids (histidine, tryptophan, tyrosine and phenylalanine) with a total concentration of 10.30 mg/100 g (1.63%). The percentage tyrosine in the total amino acids was 0.00% because tyrosine was totally absent from the amino acids profile of *Pleurotus ostreatus*.

**Table 4:** Amino acid characteristics of *Pleurotus ostreatus.*

Parameters	Ratio	Total amino acid groups	
		mg/100 g	%
Total amino acids		632.00	100
Total essential amino acids		67.831	10.733
With histidine		–	–
Without histidine		67.831	10.733
Total non-essential amino acids		564.169	89.267
Total acidic amino acids (Asp., Glu)		492.121	77.867
Total essential amino acid/Total amino acid ratio	0.11		
Total basic amino acids (Arg, lys, His)		23.182	3.668
Total basic amino acid/ Total acidic amino acid ratio	0.05	–	–
Total neutral amino acids (Gly, Ala, Leu, Ile, Val, Phe, Pro, Met, Ser, Thr, Tyr, Cys, Gln, Asn, Trp)		106.66	16.877
Total sulphur containing amino acids (Cys, Met)		19.688	3.1152
% Cys in sulphur-containing amino acids		9.3234	47.36
Total branched chain amino acids (Leu, Ile, Val)		13.567	2.1467
Total aromatic amino acids (His, Trp, Tyr, Phe)		10.3028	1.63
% Tyr in total aromatic acids		–	–

#### Mineral content of *Pleurotus ostreatus*

The mineral and heavy metal content of *Pleurotus ostreatus* is shown in Table 5. Potassium (12.25 ± 0.00 mg/kg) was the highest mineral present, followed by Iron (9.66 ± 0.00 mg/kg) and Magnesium (7.00 ± 0.00 mg/kg). Chromium was the least heavy metal present with a concentration of 0.02 ± 0.00 mg/kg.

**Table 5:** Mineral content of *Pleurotus ostreatus.*

Mineral	Concentration (mg/kg)	Recommended daily intake
Ca	0.08 ± 0.00	1,000 mg
Mg	7.00 ± 0.00	400 mg
K	12.25 ± 0.00	3,500 mg
Na	1.93 ± 0.00	2,400 mg
Zn	2.73 ± 0.00	15 mg
Fe	9.66 ± 0.00	18 mg
Pb	0.33 ± 0.00	1.0 mg
Ni	0.23 ± 0.00	1.0 mg
Cd	0.08 ± 0.00	0.036 mg
Cr	0.02 ± 0.00	0.035 mg
Na/K	0.16	1.0
Ca/Mg	0.01	2.2

Recommended daily intake [Sources: NRC (23), Afolabi et al. (24), Afolabi et al. (21), Awuchi et al. (25), and FAO (26)].

#### Vitamin profile of *Pleurotus ostreatus*

**Figure 4:**
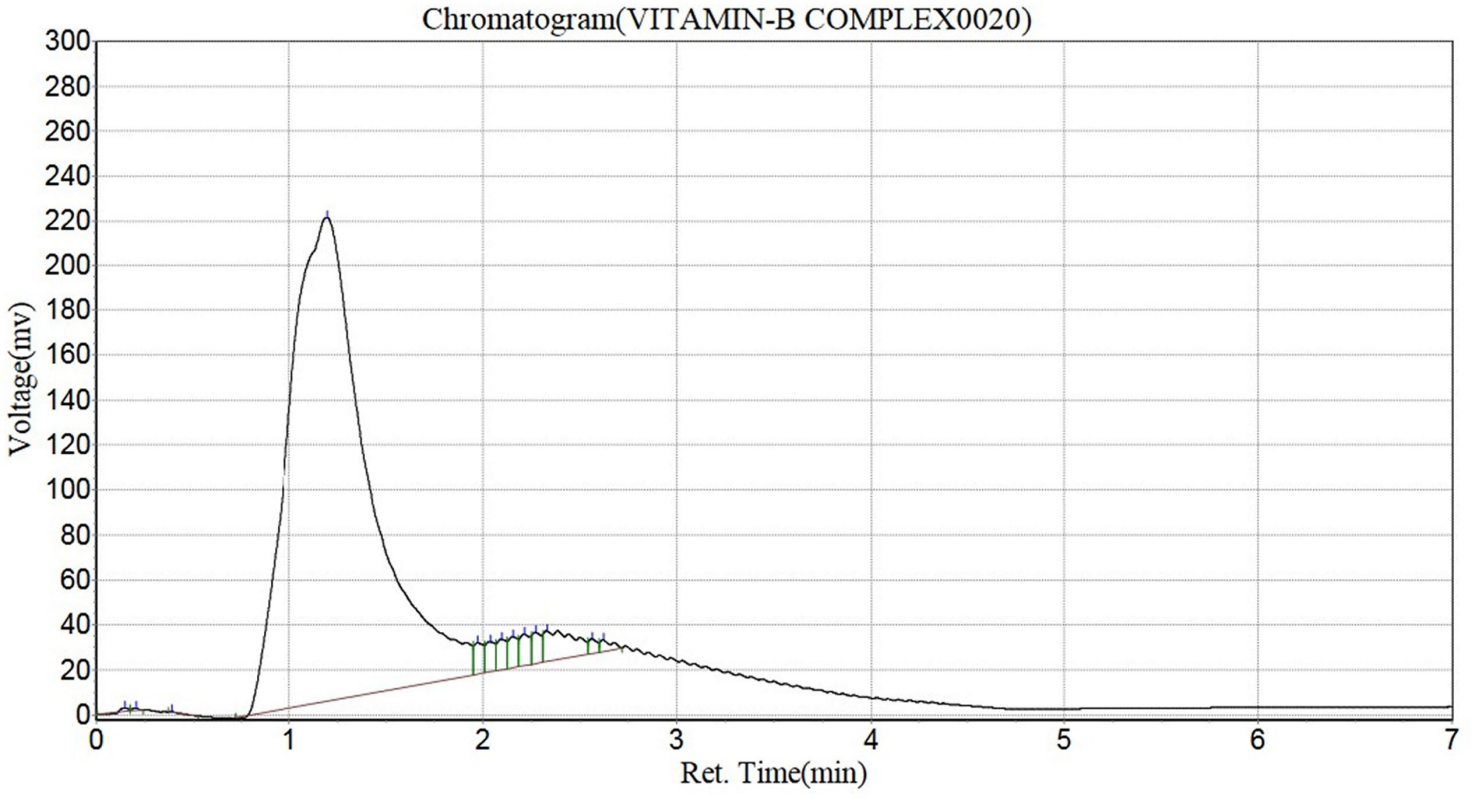
Chromatogram of the vitamin B profile in *Pleurotus ostreatus.*

The vitamin profile of *Pleurotus ostreatus* is shown in Table 6. The vitamin C content was 16.46 ± 0.12 (mg/100 g), the vitamin E content was 21.50 ± 0.14 (mg/100 g), vitamin A content was 2.93 ± 0.01 (IU/100 g). *Pleurotus ostreatus* was rich in vitamin B, with a total concentration of 99.96 mg/kg. It contained vitamin B1, B2, B3, B4, B5, B6, B7, B8, B9, B10, B11 and B12. The highest vitamin B present was vitamin B2 (riboflavin) with a concentration of 92.97 mg/kg (Figure 4).

**Table 6:** Vitamin profile of *Pleurotus ostreatus.*

Vitamin	Amount	Recommended dietary allowance
C	16.46 ± 0.12 (mg/100 g)	60 mg
E	21.50 ± 0.14 (mg/100 g)	30 (I.U.)
Beta carotene	0.06 ± 0.00 (mg/100 g)	–
A	2.93 ± 0.01 (IU/100 g)	5,000 (I.U.)
B1 (Thiamine)	0.6965 mg/kg	1.2 mg
B2 (Riboflavin)	92.9696 mg/kg	1.3 mg
B3 (Niacin)	0.7877 mg/kg	16 mg
B4 (Adenine)	0.0047 mg/kg	–
B5 (Pantothenic acid)	0.6769 mg/kg	5 mg
B6 (Pyridoxine)	0.6798 mg/kg	1.3 mg
B7 (Biotin)	2.1117 mg/kg	0.030 mg
B8 (Inositol)	0.2914 mg/kg	–
B9 (Folacin or Vit M)	0.6897 mg/kg	0.400 mg
B10 (Para-aminobenzoic acid)	0.6875 mg/kg	–
B11 (Folic acid)	0.0534 mg/kg	–
B12 (Cobalamin)	0.3107 mg/kg	0.0024 mg

Recommended Dietary Allowance [Source: Awuchi et al. (25)].

LOD – 5–25 ng/mL; LOQ – 10–27 ng/mL.

LOD, Limit of Detection; LOQ, Limit of Quantification.

## Discussion

This research highlighted the nutritional benefits of *Pleurotus ostreatus* largely as a result of the pressing demand to address the high incidence of nutrition related diseases, food insecurity and the need for readily available sources of functional foods.

### Proximate composition of *Pleurotus ostreatus*

Carbohydrates are important energy sources needed in diets (9). They are vital constituents of top quality mushrooms (27). The carbohydrate content of *Pleurotus ostreatus* was the highest (43.42%) compared to other nutrients such as protein, ash, moisture, fat and fiber. This result is in correlation with the 44.65% reported by Yusran et al. (28) for *Pleurotus ostreatus* grown in Indonesia on a wasteland consisting of majorly sawdust; 37–48% reported by Deepalakshmi and Mirunalini (29), 40.96% reported by Aguilar-Rivera and De Jesús-Merales (30) for *Pleurotus ostreatus* grown majorly on sugarcane substrate; 44.97–47.62% reported by Hoa et al. (31) for *Pleurotus ostreatus* grown on 80% saw dust in Taiwan. However, the carbohydrate content reported in this study was higher than the 5.12% reported by Elkanah et al. (32); 3.44–6.19% reported by Bernas et al. (33) and 3.33–3.37% reported by Khastini et al. (34) for *Pleurotus ostreatus* species grown on 100% sawdust. These variability show that *Pleurotus ostreatus* grown on 100% sawdust have lower carbohydrate content compared to those combined with other substances regardless of their countries of origin. Overall, the high carbohydrate content of *Pleurotus ostreatus* implies its potential as a viable source of energy in the diet which further justifies its use in making weaning food formula, breakfast meals (35), thickeners in soup, bulking agents or binders (36).

Fat serves as energy sources. It is a vital nutritional food component that helps to enhance the retaining and absorption of flavors leading to an increased food palatability. The crude fat content of the *Pleurotus ostreatus* was 1.21%. These results are in consonance with the reports of Yusran et al. (28), Elkanah et al. (32), and Irshad et al. (37). Which showed the low crude fat content of 1.51, 1.34 and 1.75%, respectively. The low crude fat content of *Pleurotus ostreatus* implies its usefulness in the management of obesity and cardiovascular diseases.

Fiber plays an important role in food substances and living organisms when consumed. They help enhance food absorption, satisfaction, prevent constipation and serve as substrates for microorganisms (27). The crude fiber content of *Pleurotus ostreatus* in this study was 23.63%. This was significantly higher than 6.72% reported by Yusran et al. (28) and 7.43% reported by Irshad et al. (37). However, it is lower than 29.75% reported by Hoa et al. (31). This variability in the fiber content despite the similarity in the nature of substrates used in various studies suggest a negligible effect of substrate type on the fiber content of *Pleurotus ostreatus*. The result on the fiber content of *Pleurotus ostreatus* in this study implies that it can be added to adult nutrition to aid in bowel movement, digestion, excretion and gut microbiome health due to its high crude fiber content. Its richness in carbohydrate and fiber, suggests its use as a prebiotic that can enhance the production of short chain fatty acids that help regulate inflammation, oxidative stress, signal transduction and boosts antioxidant profiles (38, 39). This further supports its suitability in reducing the risks of various cancer types, obesity, diabetes, cardiovascular diseases, hypertension, inflammatory bowel diseases among others (40–43).

Proteins provide amino acids which play indispensable roles in the replacement and repair of worn out cells, formation of blood proteins and boosters from the immune system (44). The protein content of *Pleurotus ostreatus* in this study was 17.06%. This is in correlation with the protein contents of 10.09–19.14%, 14.64–22.74% and 10.99–20.81% in studies by Elkanah et al. (32), Koutrotsios et al. (45), and Hoa et al. (31) respectively. This result shows that mushrooms can serve as a good protein source. It also has the potential of replacing red and processed meat protein sources (46) which contains heme iron, sulphur containing substances, mutagens that increases breast, colorectal cancer risks and other oxidative stress related diseases.

Ash is the incombustible remains of a substance. The ash content directly reflects the presence of important minerals (27). The ash contents of *Pleurotus ostreatus* in this study was 8.22%. This value is in consonance with 7.50–8.56%, 4.12–10.10 and 8.30% reported by Koutrotsios et al. (45), Elkanah et al. (32) and Aguilar-Rivera and De Jesús-Merales (30) respectively. The ash content reflects the presence of various minerals which plays diverse roles in immune regulation, maintenance of homeostasis, disease prevention and maintenance of metabolic processes (47).

Dry matter is the mass of a substance when it is contains little or no moisture, it indicates the quantity of nutrients present in a sample (48). The dry matter content of *Pleurotus ostreatus* was 8.99%. The values are comparable to the reports by Bernas et al. (33) and Zahid et al. (49) as 5.3–14.8 and 11.60%, respectively. This low dry matter content reflects that *Pleurotus ostreatus* contains high moisture compared to other nutrients (50, 51).

Moisture content refers to the amount of water present in a sample (52) Water has been known to perform numerous functions such as temperature regulation, transport function, medium for substrate dissolution and lots more (53). The moisture content of fresh and dry *Pleurotus ostreatus* was 91.01 and 6.46%, respectively. The high moisture content of fresh *Pleurotus ostreatus* shows its short shelf life and high susceptibility to microorganisms and spoilage. However, the low moisture content of the dry *Pleurotus ostreatus* signifies its less susceptibility to microbial infections and an increased shelf life span (54). The high moisture content of *Pleurotus ostreatus* also increases its significance as a functional food due to its ability to regulate metabolic processes, enhance waste removal, gut microbiome health (38) and maintenance of homeostasis (55).

#### Monosaccharide and disaccharide composition of *Pleurotus ostreatus*

The monosaccharide and disaccharide content of *Pleurotus ostreatus* is shown in Table 2. The results revealed that glucose and sucrose were the highest monosaccharide and disaccharides, respectively, present in *Pleurotus ostreatus*. This shows that *Pleurotus ostreatus* can be used as natural sweeteners, binders and food addictive (36). They are also good sources of glucose required by the body for proper functioning and energy generation. It also contains other monosaccharides and disaccharides that can serve as substrates to the gut microbiome for the production of short chain fatty acids (SCFA) which plays immunomodulatory, antioxidant and anti-cancer roles in the body (56).

#### Mineral composition of *Pleurotus ostreatus*

Minerals represent the ash content that remains after complete combustion of dry mushrooms (57). They perform vital functions in the body and are highly required for the growth and proper functioning of the body (58). *Pleurotus ostreatus* was found to contain significant amounts of K, Mg, Fe, Zn, Na and lower amounts of *Ca.* The heavy metal contents (Pb, Ni, Cd and Cr) was very low.

Calcium is a divalent cation and the most abundant element in the body which functions in neutralizing acidity, clearing toxins and building the structural framework of the body (59). They also function as cofactors to ample enzymes such as lipases, they act as an intracellular signal molecules and reduce epithelial cell proliferation in colon cancer (60). The calcium content in *Pleurotus ostreatus* (0.08 mg/kg) was low compared to the values reported by Elkanah et al. (32), Gnanwa et al. (61), Irshad et al. (37), Zahid et al. (49) as 0.19 mg/kg, 9.86 mg/kg, 3.68–5.87 mg/kg and 1.33 mg/kg, respectively. The calcium content was also lower than the recommended daily intake (RDI) of 1,000 mg (21). This signifies that *Pleurotus ostreatus* is not a very good source of calcium, however, its consumption is beneficial as it will not affect the absorption of zinc and iron from the diet.

Magnesium is the fourth most abundant mineral in the body which controls the absorption of calcium and phosphorus (62). It maintains muscle functioning and the processing of ATP, among numerous other functions (63). The magnesium content in *Pleurotus ostreatus* was 7.00 mg/kg. This value is higher than 1.40 mg and 2.65–4.60 mg reported by Irshad et al. (37) and Elkanah et al. (32) respectively. However, it was lower than 37.39 mg and 47.84 mg reported by Zahid et al. (49) and Gnanwa et al. (61). It is also significantly lower than the RDI of 400 mg, implying that a consumption of a large quantity of *Pleurotus ostreatus* is required to meet up with this requirement (64). The presence of magnesium in *Pleurotus ostreatus* increases its functionality in glucose level regulation and enzyme functioning (65).

Sodium is an important common electrolyte. It functions alongside potassium in the maintenance of fluid balance (66). The sodium content of *Pleurotus ostreatus* was 1.93 mg/kg. This is higher than 0.09–0.16 mg reported by Elkanah et al. (32) but lower than 5.31 mg and 14.50 mg reported by Zahid et al. (49) and Gnanwa et al. (61) respectively. This value is very low compared to the recommended daily intake of below 2,400 mg (21). This is equally beneficial as the low sodium content in *Pleurotus ostreatus* qualifies it to be good food substances for hypertensive patients (67).

Potassium is a common electrolyte that performs numerous critical body tasks in conjunction with sodium. It is a requirement for the maintenance of vital organs such as the heart, muscle, brain, kidney and other organs in good condition (68) with a recommended daily intake (RDI) of 3,500 mg for adults above 18 years (21). The potassium content in *Pleurotus ostreatus* was 12.25 mg/kg. This value is lower than 35.17 mg/kg reported by Zahid et al. (49) but higher than 6.70 mg, 6.67–8.78 mg and 11.98 mg reported by Gnanwa et al. (61), Elkanah et al. (32) and Irshad et al. (37) respectively. This value is low compared to the RDI. This results shows that the consumption of *Pleurotus ostreatus* can help to lower arterial blood pressure and control hypertensive and diuretic complications (65).

Iron is transition element that is ubiquitous in the biological systems. It is present in haem proteins, cytochromes, myoglobin and other enzymes (25). Iron possesses a high redox potential, interchanging from its various oxidative forms (Fe^2+^ and Fe^3+^) which is both beneficial and detrimental to the body system (69). The Iron content in *Pleurotus ostreatus* was 9.66 mg/kg. This is lower than the value reported by Irshad et al. (37) as 15.20 mg/kg but higher than 1.76 mg and 1.73–5.01 mg reported by Zahid et al. (49) and Elkanah et al. (32) respectively. This indicates that a large quantity of *Pleurotus ostreatus* is required to provide the RDI of 18 mg required by pregnant women, lactating mothers and anemic patients whose iron requirement is higher than the RDI. However, its consumption is highly suitable in cases where excess iron is to be avoided to prevent the adverse effect associated with high iron consumption that can affect the absorption of zinc, cause iron overload and serve as an initiator of free radical-mediated reactions.

Zinc is essential for health with a RDI of 15 mg/day (21). The Zinc content of *Pleurotus ostreatus* was 2.73 mg/kg. This is similar to the value reported by Elkanah et al. (32) as 2.69–2.93 mg but lower than 5.46 mg and 3–12 mg reported by Gnanwa et al. (61) and Bernas et al. (33) respectively. The moderate zinc content of *Pleurotus ostreatus* shows that when consumed it can help reduce the severity of acute diarrhea in young children and infants without interfering with the metabolism of iron and copper.

The concentration of lead, nickel, cadmium and chromium content in *Pleurotus ostreatus* was negligible and lower than the recommended daily allowance. This indicates the *Pleurotus ostreatus* despite being a saprophytic organism is safe for consumption and does not contribute to heavy metal toxicity.

#### Vitamin profile of *Pleurotus ostreatus*

Vitamins are essential chemicals substances required for the essential functioning of the body. They are needed regularly through the diet due to their importance in the growth and functioning of the body systems (25).

Vitamin A is a vital nutrient needed for vision, immune functioning and gene expression. They are good enhancers of certain minerals like zinc and Iron (70). The vitamin A content of *Pleurotus ostreatus* was 2.93 I.U./100 g. This result was higher than the reports of Majesty et al. (2) and Elkanah et al. (32) as 0.14 mg/100 g and 0.23–2.21 mg/100 g, respectively. The vitamin A content is lower than the RDA of 5,000 I.U. This indicates that a large quantity of *Pleurotus ostreatus* is required to meet up with the vitamin A RDA, however, it can be fortified to make it a better source of vitamin A. Regardless, its consumption is suitable to prevent vitamin A toxicity.

Vitamin C also called ascorbic acid is an antioxidant and a strong reducing agent (71). It increases the absorption of non-haem iron in the gastrointestinal tract. They are good enhancers of certain minerals like zinc and Iron (72). The Ascorbic acid (Vitamin C) content of *Pleurotus ostreatus* was 16.46 mg/100 g. This value is lower than 20 mg reported by Bernas et al. (33) but higher than 0.23 mg reported by Majesty et al. (2). In comparison with the 60 mg/day RNI, a large quantity of these mushrooms are needed to meet up this demand. The presence of vitamin C content in *Pleurotus ostreatus* makes it a good source of vitamin C, an exogenous antioxidant and its use in reducing oxidative stress in the body.

Vitamin E (tocopherol) majorly functions as a lipid antioxidant and maintains the integrity of the membrane. Its deficiency results in signs of membrane dysfunction (70). At high doses, it has been reported to increase the effects of vitamin A deficiency and interfere with Vitamin A absorption (2). The Vitamin E content of *Pleurotus ostreatus* was 21.50 mg/100 g. This value is higher compared to 0.09 mg/100 g reported by Majesty et al. (2). Likewise the consumption of *Pleurotus ostreatus* can supply the 30 I.U. RDA for vitamin E. *Pleurotus ostreatus* can serve as a good source of antioxidant vitamin E which can help boost immune health and reduce oxidative stress related diseases.

Mushrooms are generally good sources of B vitamins such as Thiamin (B1), Riboflavin (B2), Niacin (B3), Adenine (B4), Pantothenic acid (B5), Pyridoxin (B6), Folate, Biotin (B7), Inositol (B8), Folacin (B9), Para-aminobenzoic acid (B10), Folic acid (B11) and (Vitamin B12). These vitamins have been known to perform numerous essential functions in the provision of energy by the breakdown of certain macromolecules like fat, protein and carbohydrate (70), in the proper functioning of the nervous system, in enhancing antioxidant activities and enzymes function (25). Vitamin B2 was the highest B vitamin in *Pleurotus ostreatus*. In comparison to the RDA, Vitamin B1, B3, B5 and B6 contents were lower than their RDA of 1.2 mg/day, 16 mg/day, 5 mg/day and 1.3 mg/day, respectively. However, Vitamin B2, B7, B9 and B12 were found to higher than the RDA of 1.3 mg/day, 0.03 mg/kg, 0.40 mg/kg and 0.0024 mg/day, respectively. The vitamin B1, B2, B3 and B12 contents of *Pleurotus ostreatus* reported in this study was higher than the values reported by Majesty et al. (2) as 0.10 mg, 0.57 mg, 0.40 mg and 0.16 mg, respectively, and lower than 0.91 mg of vitamin B6. The rich vitamin B content of *Pleurotus ostreatus* enables it function in boosting the immune system, enhancing DNA synthesis (73) and improve red blood cells production, making it very suitable to be consumed by pregnant women, menstruating females and anaemic patients (74).

#### Amino acid profile of *Pleurotus ostreatus*

Amino acids are the smallest units of proteins required for the repair of worn out tissues and body building (61). Amino acids are highly required by the body for a range of functions. Based on their requirements and source, they are broadly categorized into essentials amino acids and non-essential amino acids (75). These essential amino acids are of extreme importance and must be present in required amounts in food as the body system cannot synthesize them. They include Threonine, leucine, isoleucine, lysine, methionine, phenylalanine, valine, histidine and tryptophan (76). They play roles in metabolism, immune function, formation of structural proteins, regulation of appetite, detoxification, neurotransmission, energy production, regulation of blood sugar levels, stimulation of wound healing, production of growth hormones, enzyme production and hemoglobin production (77). Table 3 shows that the highest essential amino acid present in *Pleurotus ostreatus* is lysine followed by leucine and moderate amounts of phenylalanine, methionine and threonine. This report is in contrast with the reports of Majesty et al. (2) and Gnanwa et al. (61) with leucine as the highest essential amino acid present. This signifies that *Pleurotus ostreatus* can serve as a good source of lysine and leucine.

Non-essential amino acids are readily synthesized by the body. They include alanine, aspartic acid, asparagine, glutamic acid, glutamine, glycine, proline, cysteine, serine, arginine and tyrosine (78). Aspartic acid was the highest non-essential amino acids present in *Pleurotus ostreatus*. This was followed by glutamic acid and proline which are conditionally indispensable amino acids. These amino acids play important roles in nutrition, metabolism, immune responses, anti-oxidative reactions and signaling hence reflecting the importance of the consumption of *Pleurotus ostreatus* (79).

The characterization of the amino acid profile of *Pleurotus ostreatus* is depicted in Table 4. The essential amino acid to total amino acid ratio of 0.11 reveal that *Pleurotus ostreatus* is a better source of non-essential amino acids compared to essential amino acids. This value is lower than the 0.43, 0.45 and 0.53 ratios reported by Majesty et al. (2), Bernas et al. (33), and Jin et al. (80) respectively. This value indicates that the amino acids of *Pleurotus ostreatus* may not support the synthesis of protein due to its low content of highly essential amino acids needed for protein synthesis in comparison to the overall amino acid content.

The total basic amino acid to total acidic amino acid ratio of *Pleurotus ostreatus* was 0.05, this value is less than 1, indicating that the protein is most likely acidic, hence functioning as an acid at physiological pH. It is also less than the 0.49, 0.61 and 1.03 reported by Majesty et al. (2), Jin et al. (80) and Jin et al. (80), respectively. Also, the results show that *Pleurotus ostreatus* is a better source of acidic amino acids (492.121 mg/100 g), followed by neutral amino acids (106.66 mg/kg) and least source of basic amino acids (23.182 mg/100 g). This is in contrast with the results by Majesty et al. (2), Bernas et al. (33), Jin et al. (80) which shows that *Pleurotus ostreatus* has more neutral than acidic amino acids.

Cysteine is a non-essential, sulphur containing amino acid which plays significant roles in the formation of glutathione (81). The percentage cysteine in the sulphur containing amino acids in *Pleurotus ostreatus* was 47.36% which could be attributed to the higher methionine content. The percentage cysteine content was lower than the 50% threshold expected from plants such as *Anarcardium occidentale* (50.50%) (82) and *P. africana* (60.09%), but higher than *Garcinia kola* (37.85%) (83). This implies that *Pleurotus ostreatus* might not contribute significantly to the cysteine pool when consumed. The results are similar to the percentage cysteine reported by Bernas et al. (33) as 47.62%. However, it was higher than 7.56% reported by Jin et al. (80) but lower than 73.91% reported by Majesty et al. (2).

Branched amino acids are a group of essential amino acids that possess an aliphatic side chain with a branch and serve as supplements which aid to enhance muscle growth and performance. They include leucine, isoleucine and valine (84). The percentage branched amino acids needed to meet the desired energy requirements of proteins is 10% as reported by Wardlaw and Kessel (85). However, the percentage branched amino acids content of *Pleurotus ostreatus* was 2.15% which is lower than the 10% threshold. This is also lower than the 16.08, 17.17 and 30.84% reported by Jin et al. (80), Majesty et al. (2), and Bernas et al. (33) respectively. These disparities could be as a result of the differences in the substrate used in the cultivation of the mushroom. The percentage total aromatic amino acid content in *Pleurotus ostreatus* was 1.63%. This value in was lower than 17.28% reported by Majesty et al. (2).

#### *Pleurotus ostreatus* as a functional food

The high carbohydrate content of *Pleurotus ostreatus* qualifies it to be an energy source. Its sugar profile shows that it is a good source of glucose and sucrose required by the brain and cells in the body for ATP (Adenosine Triphosphate) production (86). The diverse monosaccharides and disaccharides present, in addition to its fiber content can serve as viable substrates or prebiotics for the gut microbes to produce short chain fatty acids (SCFAs) which influence signaling pathways, contribute to antioxidants reserve and reduce inflammation (87). It also reduces cancer risk, especially breast and colon cancer where the gut bacteria play intermediary roles between the disease and its risk factors (88–90). The fiber content helps to lower blood sugar levels, making it highly beneficial for management of diabetes and numerous sugar related diseases (91). The fiber present in *Pleurotus ostreatus* also aids in increasing digestion and excretion by contributing to stool weight and reducing transit time. This has a vital significance in preventing excessive retention of wastes and reducing the contact time of toxins in feces with the walls of the intestine (90). The presence of antioxidant vitamins such as A, E and C in *Pleurotus ostreatus* qualifies it to be an exogenous source of antioxidants which can aid in free radical scavenging and oxidative stress prevention in the body (92). This ultimately helps to reduce the incidence of oxidative stress related diseases, therefore serving as a vital functional food when consumed in isolation or as a food addictive.

## Conclusion

This study evaluated the proximate, mineral, vitamins and amino acid contents of *Pleurotus ostreatus.* The proximate analysis results showed that *Pleurotus ostreatus* contained high carbohydrate and moisture contents (fresh sample of *Pleurotus ostreatus*), moderate protein and fiber content with low fat, ash and moisture contents (dry sample of *Pleurotus ostreatus*). The mineral content analysis revealed a high amount of potassium, iron and magnesium compared to other minerals. Vitamins C, E and B2 were found to be present in high amounts in the *Pleurotus ostreatus* whereas other vitamins such as A, B1, B3, B4, B5, B6, B7, B8, B9, B10, B11 and B12 were found to be present much lower concentrations. The amino acid content revealed the presence of thirteen amino acids in varying quantities. Lysine was the highest essential amino acids while aspartic acid was the highest non-essential amino acid present in *Pleurotus ostreatus*. Overall, *Pleurotus ostreatus* was found to be rich sources of nutrients, vitamins, minerals and amino acids qualifying it to be a functional food and a valuable asset in the diet to help curb the rising incidence of nutrition related diseases.

## Data availability statement

The original contributions presented in the study are included in the article/Supplementary material, further inquiries can be directed to the corresponding author.

## Author contributions

ME: Formal analysis, Investigation, Methodology, Writing – original draft, Project administration. CU: Formal analysis, Investigation, Methodology, Writing – original draft. IA: Conceptualization, Methodology, Supervision, Validation, Writing – review & editing. SC: Conceptualization, Funding acquisition, Investigation, Methodology, Project administration, Resources, Supervision, Validation, Writing – review & editing.

## References

[1] Hsieh HM Ju YM. Medicinal components in Termitomyces mushrooms. Appl Microbiol Biotechnol. (2018) 102:4987–94. doi: 10.1007/s00253-018-8991-8, PMID: 29704040

[2] Majesty DKC Winner K Univeristy R Prince O Ijeoma E. Nutritional, anti-nutritional and biochemical studies on the oyster mushroom, *Pleurotus ostreatus*. ECronicon Nutrition. (2019) 14:36–59.

[3] Achaglinkame MA Aderibigbe RO Hensel O Sturm B Korese JK. Nutritional characteristics of four underutilized edible wild fruits of dietary interest in Ghana (2019) 8:1–12. doi: 10.3390/foods8030104, PMID: 30897690 PMC6463063

[4] Ulian T Diazgranados M Pironon S Padulosi S Liu U Davies L. Unlocking plant resources to support food security and promote sustainable agriculture. Plants People Planet. (2020) 2:421–45. doi: 10.1002/ppp3.10145

[5] Lopes SO Carvalho L Abrantes S Azevedo FM. Food insecurity and micronutrient deficiency in adults: a systematic review and meta-analysis systematic review and meta-analysis. Nutrients. (2023) 15:1–14. doi: 10.3390/nu15051074 PMC10005365 36904074

[6] Beyene SD. The impact of food insecurity on health outcomes: empirical evidence from sub – Saharan African countries. BMC Public Health. (2023) 23:1–22. doi: 10.1186/s12889-023-15244-3 36793014 PMC9930357

[7] Banerjee P. Functional food: a brief overview. Int J Bioresour Sci. (2019) 6. doi: 10.30954/2347-9655.02.2019.2

[8] Reis FS Martins A Vasconcelos MH Morales P Ferreira ICFR. Functional foods based on extracts or compounds derived from mushrooms. Trends Food Sci Technol. (2017) 66:48–62. doi: 10.1016/j.tifs.2017.05.010

[9] Joshua VI Falemara BC Aina OO Courson R. Morphological characterization and proximate analysis of three edible mushrooms in plateau and Kogi states, Nigeria. World J. Microbiol. (2018) 4:139–45.

[10] Abdalla RR Ahmed AI Abdalla AI Abdelmaboud OAA Khiery NTMA Elriah NDA. Some wild edible and medicinal mushroom species at Khartoum and Sinnar States-Sudan. J Microb Biochem Technol. (2016) 8:503–6. doi: 10.4172/1948-5948.100033

[11] Fasoranti OF Ogidi CO Oyetayo VO. Nutrient contents and antioxidant properties of *Pleurotus* spp. cultivated on substrate fortified with *Selenium*. Curr Res Environ Appl Mycol. (2019) 9:66–76. doi: 10.5943/cream/9/1/7

[12] Mihai RA Melo Heras EJ Florescu LI Catana RD. The edible gray oyster fungi *Pleurotus ostreatus* (Jacq. ex Fr.) P. Kumm a potent waste consumer, a biofriendly species with antioxidant activity depending on the growth substrate. J Fungi. (2022) 8:1–19. doi: 10.3390/jof8030274, PMID: 35330276 PMC8956126

[13] Allam SFM Mohamed MO. Nutritional value, antioxidant activity and sensory evaluation of edible mushroom (*Pleurotus ostreatus*) as a supplementation to create healthier meat products. Res J Specif Educat. (2023) 1:1–25.

[14] Li H Zhang Z Li M Li X Sun Z. Yield, size, nutritional value, and antioxidant activity of oyster mushrooms grown on perilla stalks. Saudi J Biol Sci. (2017) 24:347–54. doi: 10.1016/j.sjbs.2015.10.001, PMID: 28149172 PMC5272931

[15] Mishra V Tomar S Yadav P Vishwakarma S Singh MP. Elemental analysis, phytochemical screening and evaluation of antioxidant, antibacterial and anticancer activity of *Pleurotus ostreatus* through *in vitro* and *in-silico* approaches. Meta. (2022) 12:1–25. 10.3390/metabo12090821 PMC9502197 36144225

[16] Bell V Silva CRPG Guina J. Mushrooms as future generation healthy foods. Front Nutr. (2022) 9:1–24. doi: 10.3389/fnut.2022.1050099 PMC9763630 36562045

[17] Roghini R Vijayalakshmi K. Phytochemical screening, quantitative analysis of flavonoids and minerals in ethanolic extract of *Citrus paradisis*. Int J Pharm Sci Res. (2018) 9:4859–64. doi: 10.13040/IJPSR.0975-8232.9(11).4859-64

[18] AOAC. Official methods of analysis of Association of Official Analytical Chemists, 21st edition. Arlington, Virgina, USA: AOAC (2019).

[19] Chinedu SN Nwinyi OC. Proximate analysis of *Sphenostylis stenocarpa* and *Voadzeia subterranean* consumed in south-eastern. J Agric Biotechnol Sustain Develop. (2012) 4:1–6. doi: 10.5897/JABSD11.012

[20] Sarwar S Khatun O Begum P Islam SN. Estimation of B-vitamins (B1, B2, B3 and B6) by HPLC in vegetables including ethnic selected varieties of Bangladesh. Pharm Pharmacol Int J. (2020) 8:16–23. doi: 10.15406/ppij.2020.08.00275

[21] Afolabi IS Ahuekwe EF Garuba PA Adigun AJ Afolabi IS Ahuekwe EF. *Enterococcus faecalis* -induced biochemical transformation during fermentation of underutilized *Solenostemon monostachyus* leaves *Enterococcus faecalis* -induced biochemical transformation during fermentation of underutilized *Solenostemon monostachyus* leaves. Fermentation. (2023) 9:1–20.

[22] Varinsky B Kucherenko N Kolpakova O. The research of free amino acids of water-soluble protein-polysaccharide complex of oyster mushroom *Pleurotus ostreatus*. ScienceRise. (2018) 4:32–7. doi: 10.15587/2519-4852.2018.141412

[23] National Research Council. Recommended dietary allowances. 10th ed. Wasington, DC: National Academy Press (1989).

[24] Afolabi IS Nwachukwu IC Ezeoke CS Woke RC Adegbite OA Olawole TD. Production of a new plant-based milk from *Adenanthera pavonina* seed and evaluation of its nutritional and health benefits. Front Nutr. (2018) 5:9. doi: 10.3389/fnut.2018.00009 29556498 PMC5845130

[25] Awuchi CG Victory IS Ikechukwu AO Echeta CK. Health benefits of micronutrients (vitamins and minerals) and their associated deficiency diseases: a systematic review health benefits of micronutrients (vitamins and minerals) and their associated deficiency diseases: a systematic review Awuchi. Int Peer Rev J Book Publish. (2020) 3:1–32.

[26] FAO. Human vitamin and mineral requirements, vol. 303. Rome: Food and Agriculture Organization (2001).

[27] Nasiruddin M Sultana MS Ali HFM Bodrul IM Imtiaj A. Analysis of nutritional composition and antioxidant activity of oyster mushrooms grown in Bangladesh. Int J. Food Sci Nutr. (2019) 3:223–9.

[28] Yusran Y Erniwati E Maksum H Khumaidi A Setiarto RHB. The effect of cooking on the proximate composition and minerals content of wild edible macro fungl from Lore Lindu National park, Central Sulawesi, Indonesia. Afr J Food Agric Nutr Dev. (2022) 22:20523–41. doi: 10.18697/ajfand.110.21660

[29] Deepalakshmi K Mirunalini S. *Pleurotus ostreatus*: an oyster mushroom with nutritional and medicinal properties. J Biochem Tech. (2014) 5:718–26.

[30] Aguilar-Rivera N De Jesús-Merales J. Edible mushroom *Pleurotus ostreatus* production on cellulosic biomass of sugar cane. Sugar Technol. (2010) 12:176–8. doi: 10.1007/s12355-010-0034-4

[31] Hoa HT Wang CL Wang CH. The effects of different substrates on the growth, yield, and nutritional composition of two oyster mushrooms (*Pleurotus ostreatus* and *Pleurotus cystidiosus*). Mycobiology. (2015) 43:423–34. doi: 10.5941/MYCO.2015.43.4.423, PMID: 26839502 PMC4731647

[32] Elkanah FA Oke MA Adebayo EA. Heliyon substrate composition effect on the nutritional quality of *Pleurotus ostreatus* (MK751847) fruiting body. Heliyon. (2022) 8:1–7. doi: 10.1016/j.heliyon.2022.e11841, PMID: 36468137 PMC9708699

[33] Bernas E Jaworska G Lisiewska Z. Edible mushrooms as a source of valuable nutritive constituents. Acta Scient Polonorum Technol Aliment. (2006) 5:5–20.

[34] Khastini RO Maryani N Lestari ID. Wild edible mushroom, a potential. ICSAFE. (2023) 10:160–8. doi: 10.2991/978-94-6463-090-9

[35] Hailu Kassegn H. Determination of proximate composition and bioactive compounds of the Abyssinian purple wheat. Cogent Food Agricult. (2018) 4:1–9. doi: 10.1080/23311932.2017.1421415

[36] Adebiyi AO Tedela PO Alabi OO. An inter-species comparative study on the distribution of nutrients in selected edible mushrooms in Ekiti State, Nigeria. Haya. (2018) 3:469–73.

[37] Irshad A Tahir A Sharif S Khalid A Ali S Naz A. Determination of nutritional and biochemical composition of selected *Pleurotus* spps. Biomed Res Int. (2023) 2023:1–10. doi: 10.1155/2023/8150909, PMID: 36691472 PMC9867576

[38] Törős G El-Ramady H Prokisch J Velasco F Llanaj X Nguyen DHH. Modulation of the gut microbiota with prebiotics and antimicrobial agents from *Pleurotus ostreatus* mushroom. Foods. (2023) 12:1–23. doi: 10.3390/foods12102010, PMID: 37238827 PMC10217589

[39] Zhao S Gao Q Rong C Wang S Zhao Z Liu Y. Immunomodulatory effects of edible and medicinal mushrooms and their bioactive immunoregulatory products. J Fungi. (2020) 6:1–37. doi: 10.3390/jof6040269, PMID: 33171663 PMC7712035

[40] Tsvetikova SA Koshel EI. Microbiota and cancer: host cellular mechanisms activated by gut microbial metabolites. Int J Med Microbiol. (2020) 310:151425. doi: 10.1016/j.ijmm.2020.151425, PMID: 32423739

[41] De Vos WM Tilg H Van Hul M Cani PD. Gut microbiome and health: mechanistic insights. Recent Adv Basic Sci. (2022) 71:1020–32. doi: 10.1136/gutjnl-2021-326789, PMID: 35105664 PMC8995832

[42] Weng M-T Chiu Y-T Wei P-Y Chiang C-W Fang H-L Wei S-C. Microbiota and gastrointestinal cancer. J Formosan Med Associat. (2019) 118:S32–41. doi: 10.1016/j.jfma.2019.01.002 30655033

[43] Zeppa SD Agostini D Ferrini F Gervasi M Barbieri E Bartolacci A. Interventions on gut microbiota for healthy aging. Cells. (2023) 12:1–24. 10.3390/cells12010034 PMC9818603 36611827

[44] Yang L Chu Z Liu M Zou Q Li J Liu Q. Amino acid metabolism in immune cells: essential regulators of the effector functions, and promising opportunities to enhance cancer immunotherapy. J Hematol. Oncol. (2023) 16:59–33. doi: 10.1186/s13045-023-01453-1, PMID: 37277776 PMC10240810

[45] Koutrotsios G Mountzouris KC Chatzipavlidis I Zervakis GI. Bioconversion of lignocellulosic residues by *Agrocybe cylindracea* and *Pleurotus ostreatus* mushroom fungi—assessment of their effect on the final product and spent substrate properties. Food Chem. (2014) 161:127–35. doi: 10.1016/j.foodchem.2014.03.121, PMID: 24837930

[46] González A Cruz M Losoya C Nobre C Loredo A Rodríguez R. Edible mushrooms as a novel protein source for functional foods. Food Funct. (2020) 11:7400–14. doi: 10.1039/d0fo01746a, PMID: 32896845

[47] Ali AAH. Overview of the vital roles of macro minerals in the human body. J Trace Elements Minerals. (2023) 4:100076. doi: 10.1016/j.jtemin.2023.100076

[48] Vera Zambrano M Dutta B Mercer DG MacLean HL Touchie MF. Assessment of moisture content measurement methods of dried food products in small-scale operations in developing countries: a review. Trends Food Sci Technol. (2019) 88:484–96. doi: 10.1016/j.tifs.2019.04.006

[49] Zahid MK Barua S Haque SMI. Proximate composition and mineral content of selected edible mushroom varieties of Bangladesh. Bangladesh J Nutit. (2018) 22:61–8. doi: 10.3329/bjnut.v22i0.12832

[50] Galanakis CM. Functionality of food components and emerging technologies. Foods. (2021) 10:1–26. doi: 10.3390/foods10010128, PMID: 33435589 PMC7826514

[51] Zhou Y Wang D Zhou S Duan H Guo J Yan W. Nutritional composition, health benefits, and application value of edible insects: a review. Foods. (2022) 11:3961–4002. doi: 10.3390/foods11243961, PMID: 36553703 PMC9777846

[52] Ievinsh G. Water content of plant tissues: so simple that almost forgotten? Plan Theory. (2023) 12:1–34. doi: 10.3390/plants12061238, PMID: 36986926 PMC10058729

[53] Sylvester EC Ezejiofor AJ Nnedinma EA. Survey and proximate analysis of edible mushrooms in Enugu state, Nigeria. Ann Experiment Biol. (2014) 2:52–7.

[54] Srikram S Supapvanich A. Proximate compositions and bioactive compounds of edible wild and cultivated mushrooms from Northeast Thailand. Agricult Natl Res. (2016) 50:432–6. doi: 10.1016/j.anres.2016.08.001

[55] Lorenzo I Serra-Prat M Carlos Yébenes J. The role of water homeostasis in muscle function and frailty: a review. Nutrients. (2019) 11:–15. doi: 10.3390/nu11081857, PMID: 31405072 PMC6723611

[56] Hu Y Xu J Sheng Y Liu J Li H Guo M. *Pleurotus ostreatus* ameliorates obesity by modulating the gut microbiota in obese mice induced by high-fat diet gut microbiota in obese mice induced by high-fat diet. Nutrients. (2022) 14:1–18. 10.3390/nu14091868 PMC9103077 35565835

[57] Boadu KB Nsiah-Asante R Antwi RT Obirikorang KA Anokye R Ansong M. Influence of the chemical content of sawdust on the levels of important macronutrients and ash composition in Pearl oyster mushroom (*Pleurotus ostreatus*). PLoS One. (2023) 18:e0287532–15. doi: 10.1371/journal.pone.0287532, PMID: 37384658 PMC10309632

[58] Nwozo SO Effiong ME. Phytochemical composition, mineral content and antioxidant activities of the methanol extract of *Curcuma longa* and *Viscum album*. J Food Pharmaceut Sci. (2019) 7:45–54.

[59] Jomova K Makova M Alomar SY Alwasel SH Nepovimova E Kuca K. Essential metals in health and disease. Chem Biol Interact. (2022) 367:110173. doi: 10.1016/j.cbi.2022.110173, PMID: 36152810

[60] Abd-Rabou AA. Calcium, a cell cycle commander, drives colon cancer cell diffpoptosis. Indian J Clin Biochem. (2017) 32:9–18. doi: 10.1007/s12291-016-0562-0 28149007 PMC5247361

[61] Gnanwa JM Soro LC Fagbohoun JB Yorou NS Kouame LP. Assessment of minerals, vitamins, amino and fatty acids components of Pleurotus ostreatus mushrooms cultivated and sold in the village of M'Badon (Abidjan, Côte d’Ivoire). Int J Curr Microbiol App Sci. (2021) 10:276–83. doi: 10.20546/ijcmas.2021.1009.032

[62] Farag MA Abib B Qin Z Ze X Ali SE. Dietary macrominerals: updated review of their role and orchestration in human nutrition throughout the life cycle with sex differences. Curr Res Food Sci. (2023) 6:100450. doi: 10.1016/j.crfs.2023.100450, PMID: 36816001 PMC9932710

[63] Fiorentini D Cappadone C Farruggia G Prata C. Magnesium: Biochemistry, nutrition, detection, and social impact of diseases linked to its deficiency. Nut [Internet]. (2021) 13:1–44. doi: 10.3390/nu13041136 PMC8065437 33808247

[64] Al Alawi AM Majoni SW Falhammar H. Magnesium and human health: perspectives and research directions [Internet]. Inter J Endocr Hindawi. (2018) 2018:1–17. doi: 10.1155/2018/9041694, PMID: 29849626 PMC5926493

[65] Oyetayo VO. Mineral element enrichment of mushrooms for the production of more effective functional foods (2023) 16:18–29. doi: 10.3923/ajbs.2023.18.29

[66] Salamat D Shahid MNJ. Proximate analysis and simultaneous mineral profiling of five selected wild commercial mushroom as a potential nutraceutical. Int J Chem Stud. (2017) 5:297–303.

[67] Mente A Donnell MO Yusuf S Mente A Donnell MO Yusuf S. Sodium intake and health: what should we recommend based on the current evidence? Sodium intake and health: what should we recommend based on the current evidence? Asian Food Sci J. (2020) 11:1–17.

[68] Umerah NN Nnam NM. Nutritional composition of neglected underutilized green leafy vegetables and nutritional composition of neglected underutilized green leafy vegetables and fruits in south east Geo-political zone of nigeria. Int J Chem Stud. (2017) 5:297–303.

[69] Aguirre JD Culotta VC. Battles with iron: Manganese in oxidative stress protection. J Biol Chem. (2012) 287:13541–8. 22247543 10.1074/jbc.R111.312181 PMC3340200

[70] Farhan Aslam M Majeed S Aslam S Irfan JA. Vitamins: key role players in boosting up immune response-A mini review. Vitam Miner [Internet]. (2017) 6:1–8. doi: 10.4172/2376-1318.1000153

[71] Pullar JM Carr C Vissers MC. The roles of vitamin C in skin health. Nutrients. (2017) 9:866. doi: 10.3390/nu9080866, PMID: 28805671 PMC5579659

[72] Dave KN Patil RS. Biological importance of ascorbic acid (Vitamin C) in human health – a classic review. Int J Res Biol Pharm. (2017) 3:1–8.

[73] Hrubša M Nejmanov I Vopršalov M Kujovsk L Javorsk L Mercolini L. Biological properties of vitamins of the B-complex, part 1: vitamins B1, B2, B3, and B5. Nutrients. (2022) 14:484. doi: 10.3390/nu14030484, PMID: 35276844 PMC8839250

[74] Llanaj X Törős G Hajdú P Abdalla N El-Ramady H Kiss A. Biotechnological applications of mushrooms under the water-energy-food nexus: crucial aspects and prospects from farm to pharmacy. Foods. (2023) 12. doi: 10.3390/foods12142671, PMID: 37509764 PMC10379137

[75] Leroy F Smith NW Adesogan AT Beal T Iannotti L Moughan PJ. The role of meat in the human diet: evolutionary aspects and nutritional value. Anim Front. (2023) 13:11–8. doi: 10.1093/af/vfac093, PMID: 37073319 PMC10105836

[76] Kudelka W Kowalska M Popis M. Quality of soybean products in terms of essential amino acids composition. Mol Reprod Dev. (2021) 26:1–9. 10.3390/molecules26165071 PMC8398613 34443659

[77] Kayode RMO Adedeji BS Ahmed O Aliyu TH. Screening evaluation of the nutritional composition and phytochemical of an exotic and wild species of oyster mushrooms (*Pleurotus-sajor caju*). Niger J Agric Food Environ. (2015) 33:18–21. doi: 10.1016/j.nifoj.2015.04.001

[78] Choi BH Coloff JL. The diverse functions of non-essential amino acids in cancer. Cancers. (2019) 11:675. doi: 10.3390/cancers11050675, PMID: 31096630 PMC6562791

[79] Al-Farga A Zhang H Siddeeg A Shamoon M Chamba MVM Al-Hajj N. Proximate composition, functional properties, amino acid, mineral and vitamin contents of a novel food: alhydwan (*Boerhavia elegana* Choisy) seed flour. Food Chem. (2016) 211:268–73. doi: 10.1016/j.foodchem.2016.05.016 27283631

[80] Jin Z Li Y Ren J Qin N. Yield, nutritional content, and antioxidant activity of *Pleurotus ostreatus* on corncobs supplemented with herb residues. Mycobiology. (2018) 46:24–32. doi: 10.1080/12298093.2018.1454014, PMID: 29998030 PMC6037074

[81] Fujii J Osaki T Soma Y Matsuda Y. Critical roles of the cysteine-glutathione axis in the production of γ-glutamyl peptides in the nervous system. Int J Mol Sci. (2023) 24:–28. doi: 10.3390/ijms24098044, PMID: 37175751 PMC10179188

[82] Adeyeye EL. Effect of farm and industrial processing on the amino acid profile of cocoa bean. Food Chem. (2010) 118:357–63. doi: 10.1016/j.foodchem.2009.04.127

[83] Igwe CU. Amino acid profile of raw and locally processed seeds of Prosopis Africana and ricin communis: potential antidotes to protein malnutrition. Funct Foods Health Dis. (2012) 2:107–19. doi: 10.31989/ffhd.v2i4.95

[84] Holecek M. Branched-chain amino acids in health and disease: metabolism, alterations in blood plasma, and as supplements. Nutr Metab. (2018) 15:1–12. 10.1186/s12986-018-0271-1 PMC5934885 29755574

[85] Wardlaw M Kessel C. Perspective in nutrition. 5th ed. Boston: McGraw-Hill (2002).

[86] Iheagwam FN Iheagwam OT Odiba JK Ogunlana OO Chinedu SN. Cancer and glucose metabolism: a review on Warburg mechanisms. Trop J Natl Product Res. (2022) 6:661–7.

[87] Sarma D Saha AK Datta BK. Bioactive compounds with special references to anticancer property of oyster mushroom *Pleurotus ostreatus*. J Pharmacognosy Phytochem. (2018) 7:2694–8.

[88] Hong SA Kim K Nam S Kong G Kim MK. A case – control study on the dietary intake of mushrooms and breast cancer risk among Korean women. Int J Cancer. (2008) 122:919–23. doi: 10.1002/ijc.23134, PMID: 17943725

[89] Shin A Kim J Lim S Kim G Lee E. Dietary mushroom intake and the risk of breast cancer based on hormone receptor status. Nutr Cancer. (2010) 62:476–83. doi: 10.1080/01635580903441212 20432168

[90] Song M Chan AT Sun J. Influence of the gut microbiome, diet and environment on risk of colorectal cancer. Gastroenterology. (2021) 158:322–40. doi: 10.1053/j.gastro.2019.06.048, PMID: 31586566 PMC6957737

[91] Shamim MZ Mishra AK Kausar T Mahanta S Sarma B Kumar V. Exploring edible mushrooms for diabetes: unveiling their role in prevention and treatment. Molecules. (2023) 28:2837–60. doi: 10.3390/molecules28062837, PMID: 36985818 PMC10058372

[92] Salemcity AJ Nwaneri-Chidozie VO Adameh E Eno Effiong M. Antioxidant and free radical scavenging activities of *Newbouldia laevis* leaf extracts. Free Radicals and Antioxidants. (2020) 10:10–5. doi: 10.5530/fra.2020.1.3

