# Supplementary figures and images for: Assessing the nutritional quality of Pleurotus ostreatus (oyster mushroom)
Source: Front Nutr. 2024 Jan 16;10:1279208. doi: 10.3389/fnut.2023.1279208 (PMC10824988; doi:10.3389/fnut.2023.1279208)

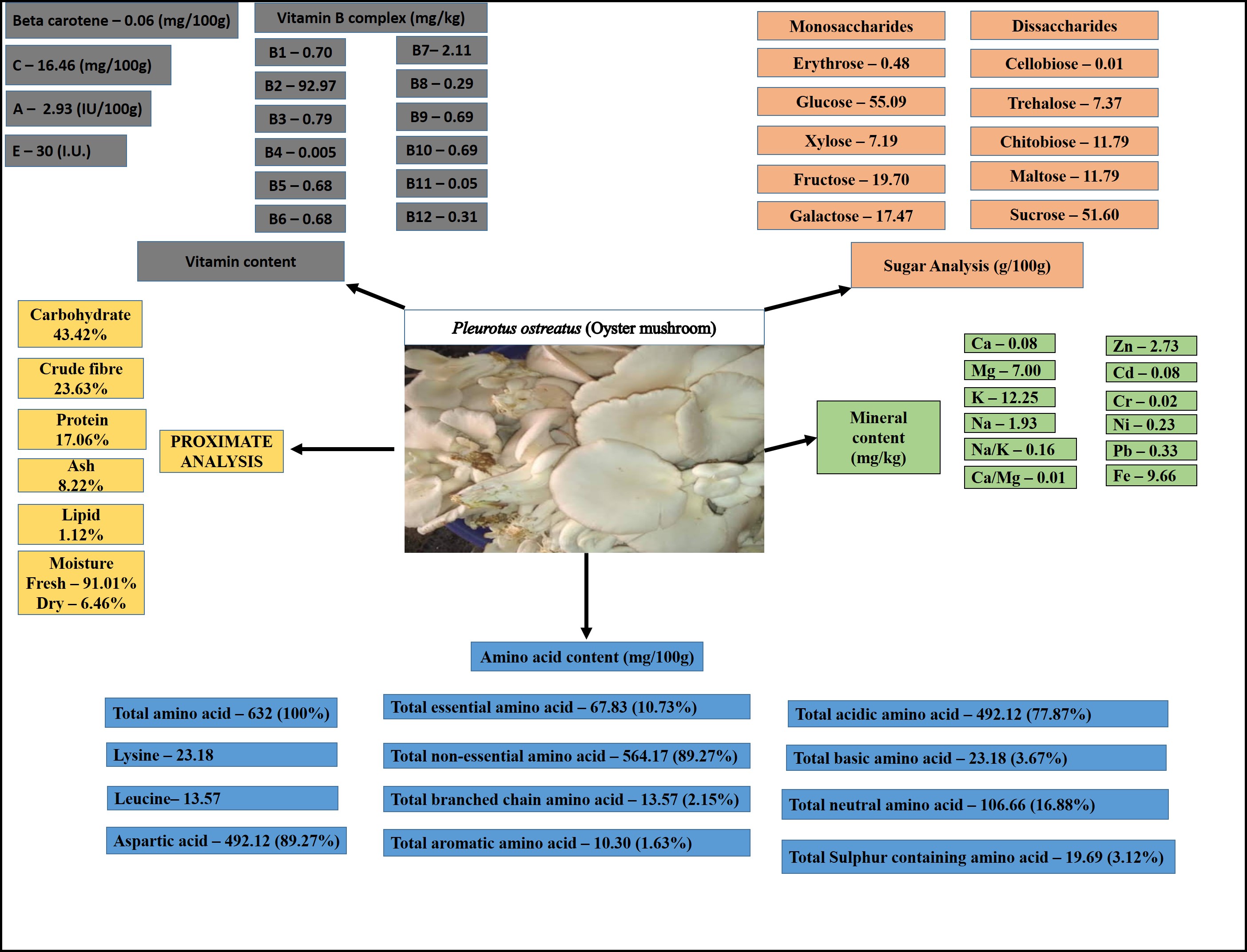

Supplement: Supplementary file 1 [file Image_1.JPEG]
